# Site-specific epitope insertion into recombinant proteins using the MAP tag system

**DOI:** 10.1093/jb/mvaa054

**Published:** 2020-05-09

**Authors:** Ayami Wakasa, Mika K Kaneko, Yukinari Kato, Junichi Takagi, Takao Arimori

**Affiliations:** m1 Laboratory of Protein Synthesis and Expression, Institute for Protein Research, Osaka University, 3-2 Yamadaoka, Suita, Osaka 565-0871, Japan; m2 Department of Antibody Drug Development, Tohoku University Graduate School of Medicine; m3 New Industry Creation Hatchery Center, Tohoku University, 2-1 Seiryo-machi, Aoba-ku, Sendai, Miyagi 980-8575, Japan

**Keywords:** antibody, epitope tag system, flow cytometry, G-protein-coupled receptor, X-ray crystallography

## Abstract

The MAP tag system comprises a 14-residue peptide derived from mouse podoplanin and its high-affinity monoclonal antibody PMab-1. We determined the crystal structure of PMab-1 complexed with the MAP tag peptide and found that the recognition required only the N-terminal 8 residues of MAP tag sequence, enabling the shortening of the tag length without losing the affinity for PMab-1. Furthermore, the structure illustrated that the MAP tag adopts a U-shaped conformation when bound by PMab-1, suggesting that loop-inserted MAP tag would assume conformation compatible with the PMab-1 binding. We inserted the 8-residue MAP tag into multiple loop regions in various proteins including fibronectin type III domain and G-protein-coupled receptors and tested if they maintain PMab-1 reactivity. Despite the conformational restraints forced by the insertion position, all MAP-inserted mutants were expressed well in mammalian cells at levels comparable to the non-tagged proteins. Furthermore, the binding by PMab-1 was fully maintained even for the mutant where MAP tag was inserted at a structurally restricted β-hairpin, indicating that the MAP tag system has unique feature that allows placement in the middle of protein domain at desired locations. Our results indicate the versatile utility of the MAP tag system in ‘site-specific epitope insertion’ application.

As recombinant DNA technology evolved, a number of protein tagging systems have been developed for various purposes such as purification, detection and quantification of recombinant proteins (1). Among the tag systems, ‘epitope tag systems’ that utilize high-affinity and high-specificity binding between a peptide tag and an anti-peptide monoclonal antibody are used in a wide range of field from cell biology to structural biology. Since a short amino-acid sequence generally <20 residues is used as a tag sequence in epitope tag systems, fusion of epitope tags have much smaller effect on the function and the folding of target molecules when compared with fusion of ‘protein tags’ such as GST tag (26 kDa), MBP tag (43 kDa) and Thioredoxin tag (12 kDa). However, attachment position of even a very short epitope tag is generally limited to either end of the target proteins (*i.e.* N- or C-terminus), since a loss of the reactivity by the monoclonal antibody or a disruption of a local conformation of a target molecule are expected when inserted in the middle of folded domain. This is because most epitope tags are recognized by their antibodies in an extended conformation and thus difficult to assume antibody-reactive conformation when positions of both ends are restrained due to the insertion topology (2–4).

The PA tag system developed by our group is one of the rare tag systems that are compatible with the insertion application. It utilizes a dodecapeptide (PA tag; GVAMPGAEDDVV) derived from human podoplanin, a type I transmembrane protein and a rat monoclonal antibody (NZ-1) against it. NZ-1 binds to the PA tag with extremely high affinity (*K*_D_ value: 4.0 × 10^−10^ M) and high specificity, and the PA tag system is applicable to various experiments including affinity purification, Western blot and flow cytometry (5). The crystal structure of the NZ-1 Fab in complex with the PA tag peptide illustrated that the peptide forms a type II β-turn in the antigen binding pocket, where both the N- and C-termini of the peptide are projecting outward from the antibody. This structural feature makes it possible to insert the PA tag into a turn-forming region of proteins without disrupting the local conformation with its affinity against NZ-1 maintained quite high (6).

The insertion compatibility of epitope tags offers several advantages over other tag systems. One benefit is that they are applicable to proteins that do not allow fusing tags to either terminal ends. The terminal regions of proteins are sometimes involved in the molecular functions or have limited accessibility from antibodies. In such cases, the ‘internal tagging’ will offer an alternative way of tag-based purification and detection. Further to that, internal tagging offers more options in choosing the tagging sites on a protein, especially when special tag location is desired due to the experimental requirement. For example, the insertion-compatible PA tag has been introduced into multiple locations of a polymorphic cell adhesion receptor αIIbβ3 integrin, enabling the detailed analysis of its conformational states during the affinity modulation (6). PA tag was also used to achieve domain/subunit identification of multi-module proteins by visualizing the inserted tag location with Fab fragment of NZ-1 antibody via electron microscopy (7, 8). Although the ‘epitope insertion’ would lead to a significant reduction in labour and cost to obtain a monoclonal antibody required for achieving specific purposes, we do not have much options for the tag systems compatible with this application other than the PA tag. Furthermore, the PA tag system has one serious drawback that precludes its application in certain experiments; due to the reactivity of the NZ-1 antibody against human podoplanin which is widely expressed on many human cell lines, it cannot be used in detecting tagged proteins expressed on human cell surface.

In the present study, we discovered that the MAP tag system is also compatible with the insertion application. The MAP tag system has been developed using a 14-residue peptide (MAP tag; GDGMVPPGIEDKIT) derived from mouse podoplanin and a rat IgG_2a_ antibody against it (PMab-1) (9, 10). PMab-1 shows high specificity and high affinity towards the MAP tag with a *K*_D_ value of 3.7 × 10^−9^ M. Similar to the PA tag system, the MAP tag system can be applied to a wide range of experiments. Although both the MAP tag and the PA tag are derived from podoplanin molecules, PMab-1 and NZ-1 show species specific podoplanin recognition and do not cross-react with podoplanin from different animals (11). This makes the MAP tag system an ideal tool to detect tagged proteins on human cell surfaces.

Here, we conducted a structural analysis of PMab-1 complexed with the MAP tag peptide by utilizing a hyper-crystallizable ‘Fv-clasp’ format developed recently in our group (12). The 2.49 Å resolution crystal structure of PMab-1 Fv-clasp revealed recognition mechanism of MAP tag peptide, suggesting that the tag sequence can be shortened to 8 residues from its original 14 residues and is compatible with the insertion into loop regions. We further demonstrated that the 8-residue MAP tag was successfully used to label cell surface G-protein-coupled receptors (GPCRs) that cannot be tagged at either termini due to the functional and topological requirements, by inserting into two short extracellular loops.

## Materials and Methods

### Preparation of PMab-1 Fv-clasp samples

Plasmids for bacterial expression of PMab-1 in the Fv-clasp(v2) format (non-tagged version), *i.e.* PMab-1V_H_(S112C)-SARAH and PMab-1V_L_-SARAH(S37C), were prepared as previously described using cDNA clones of rat anti-MAP monoclonal antibody PMab-1 (IgG_2a_, κ) isolated previously (12, 13). N43Q and N54Q mutations were introduced in the V_H_ domain of the PMab-1V_H_(S112C)-SARAH construct by a QuikChange mutagenesis, and the resultant plasmid was used for production of the PMab-1(QQ) Fv-clasp mutant in combination with the PMab-1V_L_-SARAH(S37C). Expression and purification of the Fv-clasp samples were carried out essentially according to the previously established sample preparation protocol (12), but with minor modifications. Namely, the denatured samples after the cell lysis by sonication were diluted 20-fold in modified refolding buffer (50 mM MES, 9.6 mM NaCl, 0.4 mM KCl, 2 mM MgCl_2_, 0.5 M L-arginine, 1 mM reduced glutathione and 0.1 mM oxidized glutathione) in one step. After the refolding procedure, the samples were purified using a HiLoad 16/600 Superdex 200 pg column (GE Healthcare) and a Mono Q 5/50 GL column (GE Healthcare) as previously described. To confirm the antigen binding activities of the purified samples, each sample with the amount shown in Supplementary Fig. S1C was mixed with MAP14 peptide-conjugated Sepharose and incubated for 1 h at room temperature. After washing three times with 20 mM Tris, pH 7.5 and 150 mM NaCl (Tris-buffered saline, TBS), bound proteins were eluted with sodium dodecyl sulfate-polyacrylamide gel electrophoresis (SDS–PAGE) sample buffer, subjected to 15% SDS–PAGE and stained with Coomassie Brilliant Blue.

### Structural analysis of PMab-1(QQ) Fv-clasp complexed with the MAP peptide

PMab-1(QQ) Fv-clasp was concentrated to ∼10 mg/ml by ultrafiltration using Amicon Ultra (Merck KGaA) and mixed with synthetic MAP14 peptide to be the final concentrations of 9 mg/ml PMab-1(QQ) Fv-clasp (0.24 mM) and 1 mg/ml MAP14 peptide (0.7 mM). Crystallization screening was carried out using JCSG-plus and ProPlex (Molecular Dimensions) crystallization reagents by using the sitting-drop vapour diffusion method at 20°C, and crystals appeared within a few days under 10 conditions in total. The crystals were subjected to X-ray diffraction experiment at beamline BL44XU of SPring-8 (Hyogo, Japan). One crystal grown under the condition of 0.2 M potassium formate, 20% (w/v) PEG3350 (JCSG-plus, Tube 1-10) was especially diffracted well, and diffraction data were collected at 100 K. The data were processed and scaled using X-ray Detector Software (14). Initial phase was determined by molecular replacement with PHAER (15) from the CCP4 package (16) using the crystal structures deposited in Protein Data Bank (PDB) with IDs of 5w5z, 1lk3 and 5xct as search models for V_H_, V_L_ and SARAH domains, respectively. The structural models were modified with COOT software (17) with model refinement cycle with PHENIX (18). Data collection statistics and refinement parameters are summarized in Table I.

**Table I.** Data collection and refinement statistics

	PMab-1 Fv-clasp/MAP (PDB ID: 6lz4)
Data collection	
Space group	*P*2_1_2_1_2_1_
Cell dimensions	
*a*, *b*, *c* (Å)	67.7, 85.1, 115.7
Resolution (Å)	48.15–2.49 (2.64–2.49)^a^
*R*_sym_	0.13 (1.42)
*I*/*σI*	11.55 (1.37)
CC1/2	0.998 (0.650)
Completeness (%)	99.3 (96.0)
Redundancy	8.7 (9.0)
Refinement	
Resolution (Å)	42.5–2.49
No. of reflections	23,787
*R*_work_/*R*_free_ (%)	24.5/26.9
No. of atoms	
Protein	5,081
Peptides	94
* B*-factors	
Proteins	79.8
Peptides	71.4
R.m.s. deviations	
Bond lengths (Å)	0.005
Bond angles (°)	0.830

*Note*: A single crystal was used for the structure.

a Values in parentheses are statistics of the highest-resolution shell.

### Pull-down assays

The expression construct for Fn10-Fc contained DNA segments coding for residues 1,417–1,509 of human fibronectin followed by Tobacco Etch Virus protease cleavage sequence and human IgG_1_ hinge-Fc and was made in pcDNA3.1 (Thermo Fisher Scientific)-based vector containing a bovine prolactin signal sequence. Placements of the MAP tag sequence at sites shown in Fig. 2B were conducted by extension polymerase chain reaction (PCR). Alanine-substituted mutants of Fn10-Fc were prepared by QuikChange strategy. Each plasmid was transiently transfected into Expi293F cells (Thermo Fisher Scientific) according to the manufacturer’s protocol. Culture supernatants were harvested 4 days after the transfection and subjected to the pull-down assays using PMab-1-immobilized Sepharose or rProtein A Sepharose (GE Healthcare). After washing three times with TBS, bound proteins were eluted from the beads with SDS–PAGE sample buffer, subjected to 12.5% SDS–PAGE and stained with Coomassie Brilliant Blue.

### Flow cytometry

DNA segments coding for protease activated receptors (PARs), *i.e.* residues 36–426 of PAR1 and residues 25–385 of PAR4, were cloned into a phCMV3 (Genlantis)-based vector containing a bovine prolactin signal sequence and a N-terminal FLAG tag. The MAP tag was inserted into sites shown in Fig. 3B by inverse PCR. Expi293F cells were transiently transfected with each plasmid. Cells were harvested after 48 h of transfection and incubated with the anti-FLAG M2 antibody (Sigma-Aldrich, F3165) diluted at 1:500 in Dulbecco’s Modified Eagle Medium (DMEM) with 10% foetal calf serum (FCS) or 1.0 μg/ml PMab-1 in DMEM/FCS for 2 h on ice. The cells were then washed twice with DMEM/FCS and resuspended in DMEM/FCS containing Alexa-Fluor-488-conjugated goat anti-mouse IgG (Thermo Fisher Scientific, A11029) diluted at 1:500 (for M2) or Alexa-Fluor-488-conjugated goat anti-rat IgG (Thermo Fisher Scientific, A11006) diluted at 1:500 (for PMab-1). After 30 min incubation on ice, the cells were washed twice and suspended in phosphate-buffered saline (pH 7.4) and analysed on a flow cytometer EC800 system (Sony). The data were analysed with FlowJo software (BD).

## Results

### Crystallographic analysis of the PMab-1 Fv-clasp in complex with the MAP tag peptide

In order to facilitate the crystallization of PMab-1, we first converted it to a small antibody fragment format ‘Fv-clasp’ developed recently (12). Fv-clasp is a fusion of an anti-parallel coiled-coil structure derived from the human Mst1 SARAH domain to the antibody variable region (*i.e.* V_H_ and V_L_ domains) and known to exhibit very high crystallization tendency compared to the corresponding Fab fragment. Additionally, we mutated two asparagine residues (Asn43 and Asn54) in the V_H_ domain to glutamine [PMab-1(QQ) Fv-clasp] because they constitute ‘Asn-Gly’ sequences. The asparagine residue in this sequence is prone to the spontaneous deamidation reaction under high-pH and high-temperature conditions, introducing a chemical heterogeneity in the crystallization sample (19). We confirmed that the mutation eliminated the appearance of the deamidated protein peak in the anion exchange chromatography, without affecting the apparent antigen binding activity (Supplementary Fig. S1).

**Fig. 1.**
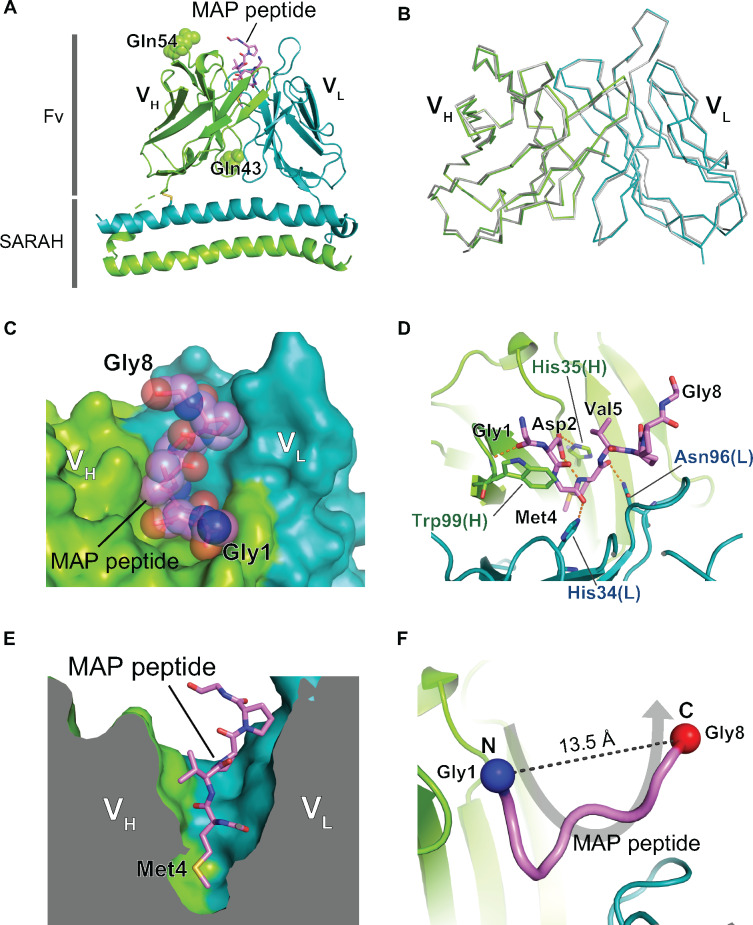
**Crystal structure of the PMab-1(QQ) Fv-clasp in complex with the MAP tag peptide.** (**A**) Overall structure of the PMab-1(QQ) Fv-clasp-MAP tag peptide complex. The V_H_-SARAH and the V_L_-SARAH are shown as cartoon presentation. The bound MAP tag peptide is shown as a stick model. The two substituted Gln residues, Gln43 and Gln54, in the V_H_ domain are shown as sphere models. (**B**) Comparison of the Fv structures between the two PMab-1(QQ) Fv-clasp molecules in the asymmetric unit. The Fv region of the Mol-2 (grey) is superposed on that of the Mol-1 (coloured), and they are shown in Cα tracing. (**C** and **D**) The expanded views of the antigen binding pocket of PMab-1. PMab-1 is shown as a surface model, and the MAP tag peptide is shown as a stick model with a transparent sphere model (C). PMab-1 residues involved in the MAP tag peptide recognition are shown as stick models, and hydrogen bonds are denoted by dashed lines (D). (**E**) Sliced-surface view at the antigen binding pocket. Note that the side chain of Met4 of the MAP tag peptide is deeply inserted into a cavity formed between the V_H_ and the V_L_. (**F**) U-shaped conformation of the MAP tag peptide bound by PMab-1. The MAP tag peptide is presented as a worm model. The Cα atoms of Gly1 and Gly8 are shown as sphere models.

Crystallization screening of the PMab-1(QQ) Fv-clasp was carried out in the presence of excess amount of the 14-residue MAP tag peptide, and crystals appeared under many conditions. The crystals obtained in this initial screening were directly subjected to X-ray diffraction experiments without further optimization, and one of the crystals diffracted to 2.49 Å resolution (Table I). The crystal contained two PMab-1(QQ) Fv-clasp molecules in the asymmetric unit (Mol-1 and Mol-2), and extra electron densities were found in the antigen binding pocket of both molecules after model building of the Fv-clasp region. We carefully analysed the electron densities and finally assigned the peptide sequence from the N-terminal 8 and 7 residues into the densities of Mol-1 and Mol-2, respectively (Fig. 1A and Supplementary Fig. S2). The Fv region structure of the two molecules in the asymmetric unit is nearly identical, with root-mean-square deviation value of 0.47 Å for superposed 210 Cα atoms, and notable difference was not observed even in the antigen binding sites (Fig. 1B). Because the overall electron density of Mol-1 is slightly better than that of Mol-2, we will use the Mol-1 model in the following discussion unless otherwise specified.

The MAP tag peptide fits into the PMab-1 antigen-binding groove formed between the V_H_ and V_L_ domains (Fig. 1C). The substituted glutamine residues (Gln43 and Gln54) are located far from the antigen binding site, consistent with the lack of functional effect of these mutations (Fig. 1A and Supplementary Fig. S1C). Unlike typical antigen binding mode of sequence-specific anti-peptide antibodies, the peptide recognition by residues in the complementarity-determining region (CDR) of PMab-1 involved many interactions with the main chain portion of the MAP tag peptide (Fig. 1D), except for the side chains of just two residues, Asp2 and Met4. The side chain of Asp2 forms an intermolecular hydrogen bond with the side chain of His35 in the CDR-H1 of PMab-1 (Fig. 1D). The side chain of Met4 is completely buried in the antibody molecule by inserting it into the hydrophobic cave at the centre of the antigen-binding groove (Fig. 1E). Furthermore, Asp2 side chain makes an intramolecular hydrogen bond with the main chain amide of Met4, stabilizing the compact conformation of the Asp-Gly-Met tripeptide motif to ensure its accommodation in the small binding pocket. These observations strongly suggest that Asp2 and Met4 but not other residues in the MAP tag play critical roles in the sequence-specific binding by PMab-1. Another intriguing observation in the crystal structure is that the MAP tag peptide adopts a U-shaped conformation where the both terminal ends are exposed to solvent (Fig. 1F), suggesting that the MAP tag is compatible with the insertion into loop regions as in the case of the PA tag.

### Optimization of the MAP tag sequence in length

**Fig. 2.**
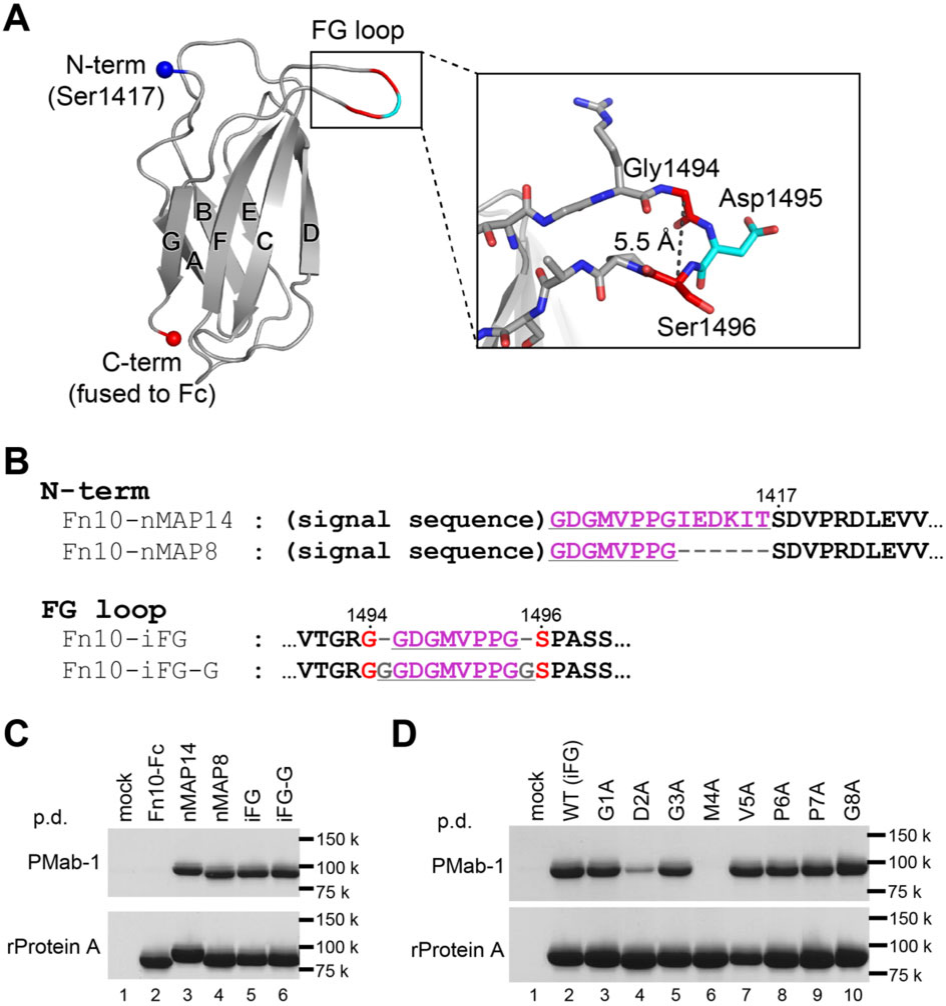
**The MAP tag can be inserted into the long protruded loop of Fn10.** (**A**) Fn10 structure extracted from the crystal structure of human fibronectin fragment (PDB ID: 1fnf) is shown as a cartoon model. The N- and C-termini of Fn10 are shown as spheres. The seven strands forming the β-sandwich structure of Fn10 are labelled with A–G. The expanded view of the FG loop shown as stick models is provided in the inset. (**B**) The amino-acid sequences for N-terminally MAP-tagged or MAP-inserted Fn10-Fc mutants near the MAP tag-fused portions. The MAP tag-derived sequences and linker residues are underlined. Residue numbers of original Fn10 are provided above the sequences. (**C** and **D**) Pull-down assay of the MAP-tagged Fn10-Fc by PMab-1-immobilized Sepharose (upper panel) and rProtein A Sepharose (lower panel). The Fn10-Fc samples were expressed in Expi293F cells and precipitated with each resin, followed by 12.5% SDS–PAGE under non-reducing conditions and stained with Coomassie Brilliant Blue. All mutants were expressed well in Expi293F cells as confirmed by the rProtein A Sepharose pull-down assay (C, D, lower panels). Note that the nMAP8, iFG and iFG-G samples were captured by PMab-1 with equivalent efficiency with the nMAP14 sample (C, top panel) and that the D2A and M4A mutants showed severe decrease in binding affinity for PMab-1 (D, top panel).

The fact that the C-terminal half of the MAP tag peptide was disordered and not involved in the recognition by PMab-1 points to the possibility that the MAP tag sequence can be shortened without reducing the affinity towards PMab-1. From the structure, the first 7 residues are expected to be essential for the recognition. As to the Gly8, corresponding electron density was of poor quality (in Mol-1) or invisible (in Mol-2), so it is possible that Gly8 has little or no contribution to the PMab-1 binding (Supplementary Fig. S2). Nevertheless, we chose the 8-residue portion as the new shortened version of MAP tag (MAP8 tag; GDGMVPPG), hoping that the last Gly may contribute favourably to the recognition by functioning as a spacer. First, we assessed the function of the MAP8 tag by attaching it at the N-terminal of a model protein, the tenth fibronectin type III domain of human fibronectin (Fn10). Fn10 is a well-folded small protein (10 kDa) with a β-sandwich structure composed of seven strands and is frequently used as a scaffold for studies on protein engineering, biophysics and protein folding (Fig. 2A) (20, 21). For efficient expression in mammalian cells and easy detection by the pull-down assays, we fused the human IgG_1_ Fc domain to the C-terminus of Fn10 (called Fn10-Fc hereafter). Either the MAP8 or the original 14-residue MAP tags were fused to the N-terminus of Fn10-Fc (Fn10-nMAP8 and Fn10-nMAP14; Fig. 2B). The proteins were expressed in Expi293F cells, and their expression levels and PMab-1 reactivities were analysed by the pull-down assay from the conditioned media using rProtein A Sepharose and PMab-1-immobilized Sepharose, respectively. Fn10-Fc forms a dimer via disulphide bonds and migrates as a single band of ∼90 kDa in non-reducing SDS–PAGE gel. As shown in Fig. 2C, lanes 2–4, Fn10-nMAP8 showed binding ability comparable to Fn10-nMAP14 against PMab-1, indicating that the 8-residue MAP tag sequence is long enough for the full PMab-1 binding.

### Insertion of the MAP8 tag into an extended loop region of Fn10

Next, we tested if the MAP8 tag can be inserted into a loop region of a protein. The long and protruding FG loop of Fn10 was chosen as an insertion point because it is known to tolerate various sequence modifications (Fig. 2A) (22, 23). We designed a mutant of Fn10-Fc by inserting the MAP8 tag between Gly1494 and Ser1496 after removing Asp1495 located at the tip of the FG loop (Fn10-iFG8, Fig. 2B). In the MAP–PMab-1 complex structure, the distance between the Gly1 and Gly8 is 13.5 Å (Fig. 1F). As the Cα atoms of Gly1494 and Ser1496 are separated by only 5.5 Å in the Fn10 structure (PDB ID: 1fnf; Fig. 2A), we thought that the MAP8 insertion may cause disruption of the local conformation of Fn10 and decrease the binding affinity of the inserted tag towards PMab-1. Thus, we included another mutant where the inserted MAP8 was flanked by extra Gly residues at both ends (Fn10-iFG-G, Fig. 2B). Both Fn10-iFG and Fn10-iFG-G were expressed well at levels equivalent to the non-tagged Fn10-Fc, indicating that the insertion of the MAP tag sequence did not affect the Fn10 structure (Fig. 2C, bottom, lanes 2, 5 and 6). Moreover, as judged by the pull-down assay using PMab-1-immobilized Sepharose, bindings of both MAP8-insertion mutants were indistinguishable from that of N-terminally MAP-tagged samples (Fig. 2C, top, lanes 3–6), indicating that the MAP8 tag inserted at a loop remains fully functional even without any linker residues, as long as the loop is long enough.

### Alanine scanning of the MAP tag sequence

For a detailed investigation of the MAP tag recognition mechanism by PMab-1, each residue in the MAP8 tag sequence was individually mutated to alanine (G1A, D2A, G3A, M4A, V5A, P6A, P7A and G8A mutants) in the context of Fn10-iFG, and the binding of the mutants to PMab-1 was evaluated by the pull-down assay. As a result, the D2A mutant showed a marked decrease in the binding activity to PMab-1, and the M4A mutant completely lost the reactivity (Fig. 2D). On the other hand, none of other mutants showed appreciable decrease in the binding. This result is fully consistent with the structural analysis of the complex described earlier, and agrees with the previous study that evaluated the PMab-1 binding towards Ala-substituted full-length mouse podoplanin expressed on cell surface by flow cytometry (24). Thus, we concluded that Asp2 and Met4 in the MAP tag sequence are irreplaceable residues in this tag system.

### MAP8 tag insertion into the surface-exposed loop of GPCRs

**Fig. 3.**
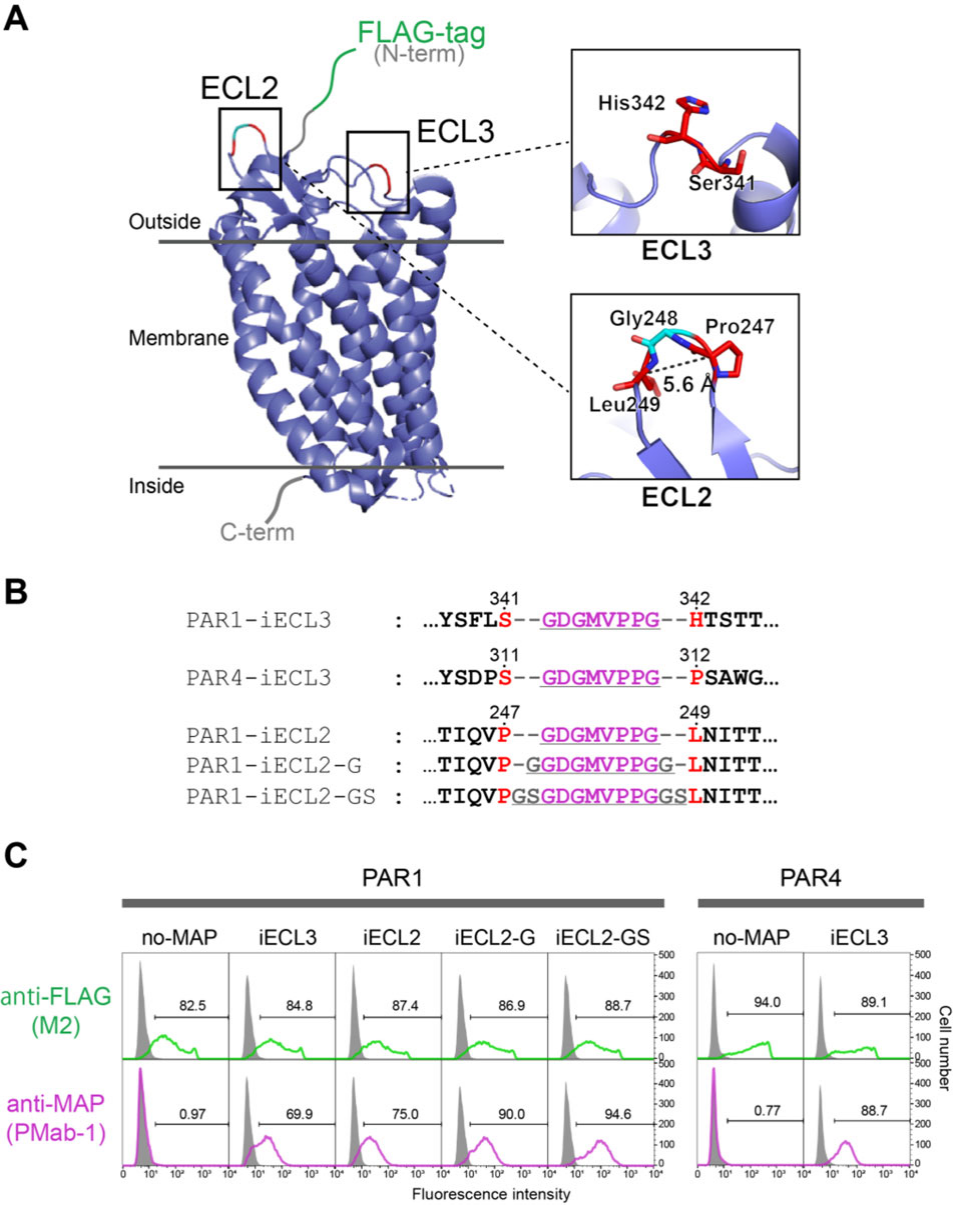
**The MAP-inserted GPCRs expressing on human cells were efficiently stained with PMab-1 in flow cytometry analysis.** (**A**) Overall structure of PAR1. The crystal structure of PAR1 was determined as a T4 lysozyme-fused mutant (PDB ID: 3vw7), and the figure was drawn as a cartoon model after eliminating the T4 lysozyme portion from the model. The expanded view at the MAP tag insertion regions is shown in the right. (**B**) The amino-acid sequences for MAP-inserted PAR1 and PAR4 mutants near the insertion portions. The MAP8 tag sequence and linker residues are underlined. Residue numbers of the original receptors are provided above the sequences. (**C**) Flow cytometry analysis of MAP-inserted PAR1 and PAR4 mutants. PAR1 and PAR4 mutants were transiently expressed on Expi293F cells and incubated with PMab-1 or anti-FLAG antibody M2, followed by staining with corresponding Alexa-Fluor-488-labelled secondary antibodies. Signals from cells transfected with each sample and not treated with any antibodies (grey area) were used to define PMab-1- or FLAG-positive cell populations (regions including <1% of non-treated cells were indicated by the brackets and defined as the positive region for each antibody). Staining efficiencies are expressed as the % positive cells and provided in the each panel.

The successful insertion of the MAP8 tag into the protruding loop of Fn10 implies that the inserted MAP tag formed the U-shaped conformation as observed in the crystal structure without disrupting the conformation of Fn10. This may not be surprising, however, because Fn10 is stable enough to withstand artificial modifications at the FG loop. To demonstrate that the MAP8 tag has broad utility in the loop-insertion applications, we chose GPCRs, the largest family of membrane receptors that present major challenges during the expression, purification and structural analyses for drug discovery, as the insertion target. GPCRs are seven-transmembrane receptors that have three extracellular loops (ECL1–3) and three intracellular loops (ICL1–3) within the molecules. The C-terminus of GPCRs is located on the cytoplasmic side where antibodies cannot access from outside the cell. Therefore, a tag should be fused to the extracellular N-terminal side when detection of cell surface GPCRs is required. However, the N-terminal region of certain GPCRs cannot be modified for functional reasons. Such case is exemplified by the GPCR subclass called PARs. Two representative PARs, PAR1 and PAR4, are activated by a proteolytic digestion at the N-terminal region (*i.e.* between Arg41 and Ser42 of PAR1 and between Arg47 and Gly48 of PAR4) by thrombin, and the newly exposed N-termini after the cleavage behave as tethered ligands that activate the receptors for the downstream signalling (Supplementary Fig. S3) (25). Because of this nature, the N-terminal tagging cannot be accepted in PARs, especially when the post-cleavage receptor is to be analysed. We decided to test MAP8’s insertion utility by using PAR1 and PAR4 as the target proteins. The crystal structure of PAR1 but not PAR4 is available (Fig. 3A) (26). In PAR1, the ECL3 connecting the C-terminus of the transmembrane helix 6 and the N-terminus of the transmembrane helix 7 is rather long and largely exposed, especially at the side chains of Ser341 and His342 (Fig. 3A). We first tested if the MAP8 tag can be inserted into this region of PAR1 (PAR1-iECL3) and the corresponding site of PAR4 (between Ser311 and Pro312, PAR4-iECL3) (Fig. 3B and Supplementary Fig. S3). Since the biological activities of the receptors were not to be evaluated in this experiment, we fused the FLAG tag to the N-terminus of all samples as a control tag to monitor the expression profiles. The receptors were expressed on the surface of Expi293F cells and analysed for the PMab-1 reactivity by flow cytometry. As shown in Fig. 3C, upper panels, expression profiles of the MAP-inserted receptors were essentially the same as those of their respective control receptors that does not have the MAP tag, suggesting that the MAP tag insertion did not grossly affect the structural integrity of these GPCRs. More importantly, MAP8 tag inserted into the ECL3 was recognized well by PMab-1, as evident from the strong staining of cells transfected with these receptors (Fig. 3C, lower panels). It is particularly notable that even the MAP8 tag inserted into ECL3 of PAR4, which was designed without an experimental structure of PAR4, was efficiently recognized by PMab-1, indicating that the precise structural information is not always necessary for designing MAP tag insertion constructs.

We next investigated if the MAP tag can be inserted into more structurally restricted region, a β-hairpin. ECL2 of PAR1 assumes a β-hairpin structure with the distance between the Cα atoms of Pro247 and Leu249 being 5.6 Å (Fig. 3A). In contrast to the long and mobile Fn10 FG loop, the β-hairpin of the ECL2 would force close apposition of the termini of the MAP8 peptide when inserted, which may result in the conformation incompatible with the PMab-1. Therefore, we inserted MAP8 tag between Pro247 and Leu249 of PAR1 with 0, 1 and 2 spacer residues at both ends (Fig. 3B). Again all mutants were expressed well regardless the linker length (Fig. 3C, upper panels) and, surprisingly, all insertion mutants including the plain MAP8 insertion (*i.e.* no spacers) were recognized by PMab-1 (Fig. 3C, lower panels), indicating that the MAP8 tag retains high reactivity to PMab-1 even when inserted into a very tight hairpin of GPCRs.

## Discussion

In the present study, we showed that the MAP tag shortened to 8 residues (*i.e.* MAP8 tag) is fully functional and Asp2 and Met4 of the MAP8 tag are critical for the binding. It is noteworthy that all residues consisting the MAP8 tag except for Asp2 and Met4 are small amino acids. In our crystal structure, the MAP8 tag peptide fits tightly in the small antigen binding pocket of PMab-1, and there is no room to accommodate larger amino acids in the pocket (Fig. 1C). Therefore, the MAP tag seems to achieve its high sequence specificity by using many ‘featureless’ amino acids rather than an elaborate combination of large side chains, which is typical for many anti-peptide antibodies. Although only a short segment of the MAP8 tag is recognized by PMab-1, all residues are efficiently used to constitute the binding epitope that spans relatively large contact area, which warrants high affinity. In fact, the contact area on the 7-residue MAP tag (excluding the Gly8 that does not seem to contribute much to the interface) is 661.0 Å^2^, accounting for ∼75% of the total surface area of the entire peptide, which is comparable to that between the 9-residue HA tag and its monoclonal antibody 12CA5 (675.7 Å^2^, which corresponds to 49% of the total surface area of the HA peptide). In addition, the relatively low total chemical complexity of the amino acids found in the MAP8 tag sequence is expected to have less deteriorating effects on the functional and physical properties of target molecules.

The most important finding in this study is the fact that the MAP8 tag can be inserted into loop regions of proteins. Since small difference in PMab-1 binding efficiency was observed among the PAR1-iECL2 mutants depending on the linker length (Fig. 3C), linker optimization may be sought for each insertion design to achieve the maximum reactivity when inserting the MAP8 tag into loops. Nevertheless, it is also notable that all MAP-inserted mutants tested in this study showed sufficient sensitivity to PMab-1 for detection even without any linker. As we did not see difference in the PMab-1 reactivity against terminally added MAP tag and inserted ones (Fig. 2C), the affinity difference between the two different tag presentation modes seems to be negligible, as long as they remain fully accessible to the antibody. This loop-insertion compatibility of the MAP tag system expands its utility, especially in the structural biology field. In X-ray crystallography, antibody fragments are often used as crystallization chaperones which facilitate the crystallization of target molecules by improving structural homogeneity and providing lattice packing interactions (27–29). As monoclonal antibodies that reduce structural flexibility upon binding to the native three-dimensional epitope on the target molecule must be used in the crystallization chaperone application; however, it is generally necessary to screen for large number of available antibodies or prepare new antibody for this purpose only. Recently, Tamura *et al.* succeeded in utilizing the PA tag system as a crystallization chaperone, by inserting the PA tag into a β-hairpin region of a bacterial Site-2 protease and making complex with anti-PA NZ-1 antibody Fab fragment. The resolution of the diffraction data from the NZ-1-bound PA-inserted sample was improved to 2.0 Å compared to the native (*i.e.* non-tagged) sample that diffracted to 2.8 Å resolution (30), suggesting that the bound Fab assisted the formation of the well-ordered crystals. Thus, we expect that the ‘epitope insertion’ or ‘epitope grafting’ may become a popular method in the chaperone-assisted crystallography, because the establishment of a monoclonal antibody for each target molecule is no longer necessary. Furthermore, this method can greatly simplify the experimental workflow because both protein purification and chaperone-assisted crystallization are achieved with a single tag system, and the enzymatic treatment for removing the tag before crystallization can be omitted.

The ‘epitope grafting’ would also be useful for high resolution structural analysis via cryo-electron microscopy (cryo-EM). Due to the recent advances in the cryo-EM methodology, determination of near-atomic resolution three-dimensional structures of macromolecules that are biologically important and difficult to crystallize is reported one after another. However, high resolution cryo-EM analysis of structurally flexible, small in size (<100 kDa) and unstable samples is still considered as a challenging task. To address these problems, many researchers use antibody fragments with the hope of improving structural homogeneity, increasing size and improving stability of target molecules (31–35). The bound antibody fragments also serve as a fiducial marker to facilitate particle alignment. Therefore, PMab-1 Fv-clasp or Fab fragment bound to the grafted MAP8 tag can also facilitate cryo-EM analysis of difficult targets.

GPCRs are famous molecules that often require assistance of antibody fragments in their structural analysis due to the hydrophobic nature unfavourable for the crystallization and the small molecular size unfavourable for cryo-EM observation in addition to the structural flexibility. Difficulty in sample preparation of GPCRs with sufficient amount and purity for structure determination is also a troubling issue, and the tag systems are essential for exploring expression constructs suitable for structural analysis and achieving efficient protein purification. Therefore, our result that the MAP8 tag can be inserted into the loop regions of PAR1 and PAR4 strongly suggests that it can be an extremely useful tool for high-throughput structure determination of challenging targets including GPCRs.

## Supplementary Data

Supplementary Data are available at *JB* Online.

## Supplementary Material

mvaa054_supplementary_data

## Abbreviations

CDR: complementarity-determining region
cryo-EM: cryo-electron microscopy
PCR: polymerase chain reaction
TBS: Tris-buffered saline
SDS-PAGE: sodium dodecyl sulfate-polyacrylamide gel electrophoresis
DMEM: Dulbecco’s Modified Eagle Medium
FCS: foetal calf serum
GPCR: G-protein-coupled receptor
PAR: protease activated receptor
PDB: Protein Data Bank.
