# Supplementary material for: Site-specific epitope insertion into recombinant proteins using the MAP tag system
Source: J Biochem. 2020 May 9;168(4):375–84. doi: 10.1093/jb/mvaa054 (PMC7585734; doi:10.1093/jb/mvaa054)
Supplement: mvaa054_supplementary_data [file mvaa054_supplementary_data.pdf]

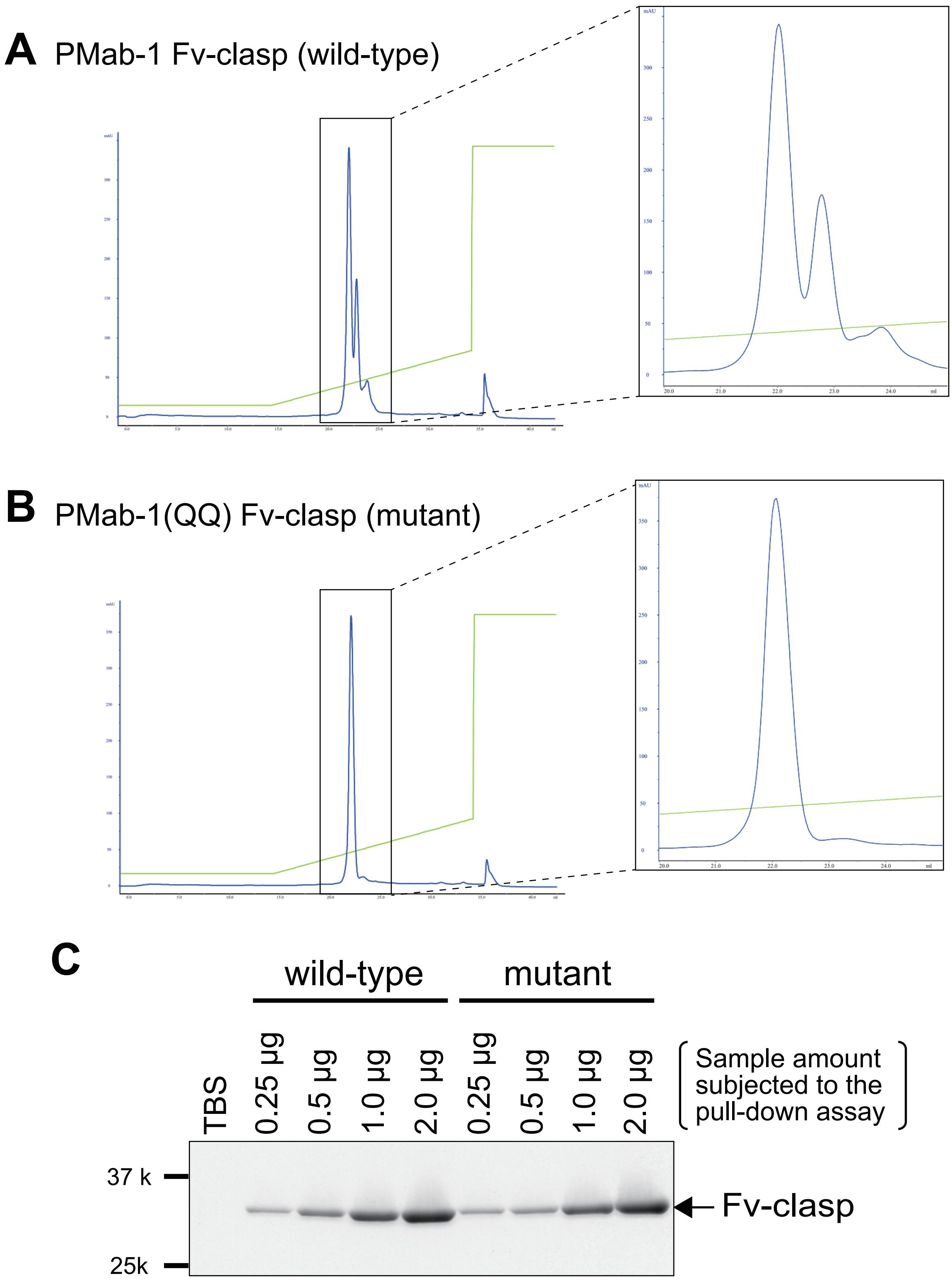

Supplementary Fig. S1 The PMab-1 Fv-clasp(QQ) mutant shows improved sample homogeneity and retains high antigen-binding activity. (A and B) Anion-exchange chromatography profiles of PMab-1 Fv-clasp (A) and PMab-1(QQ) Fv-clasp (B). Note that the wild-type sample eluted in several peaks, while the mutant eluted in a single peak, indicating that the sample homogeneity of PMab-1 Fv-clasp was improved by the mutations. (C) Pull-down assay by MAP peptide-conjugated Sepharose. The purified Fv-clasp samples with the amounts indicated above each lane were subjected to the pull-down assay. The mutant sample showed band intensities comparable to the wild-type sample, confirming that mutant retains sufficient binding activity for the MAP peptide.

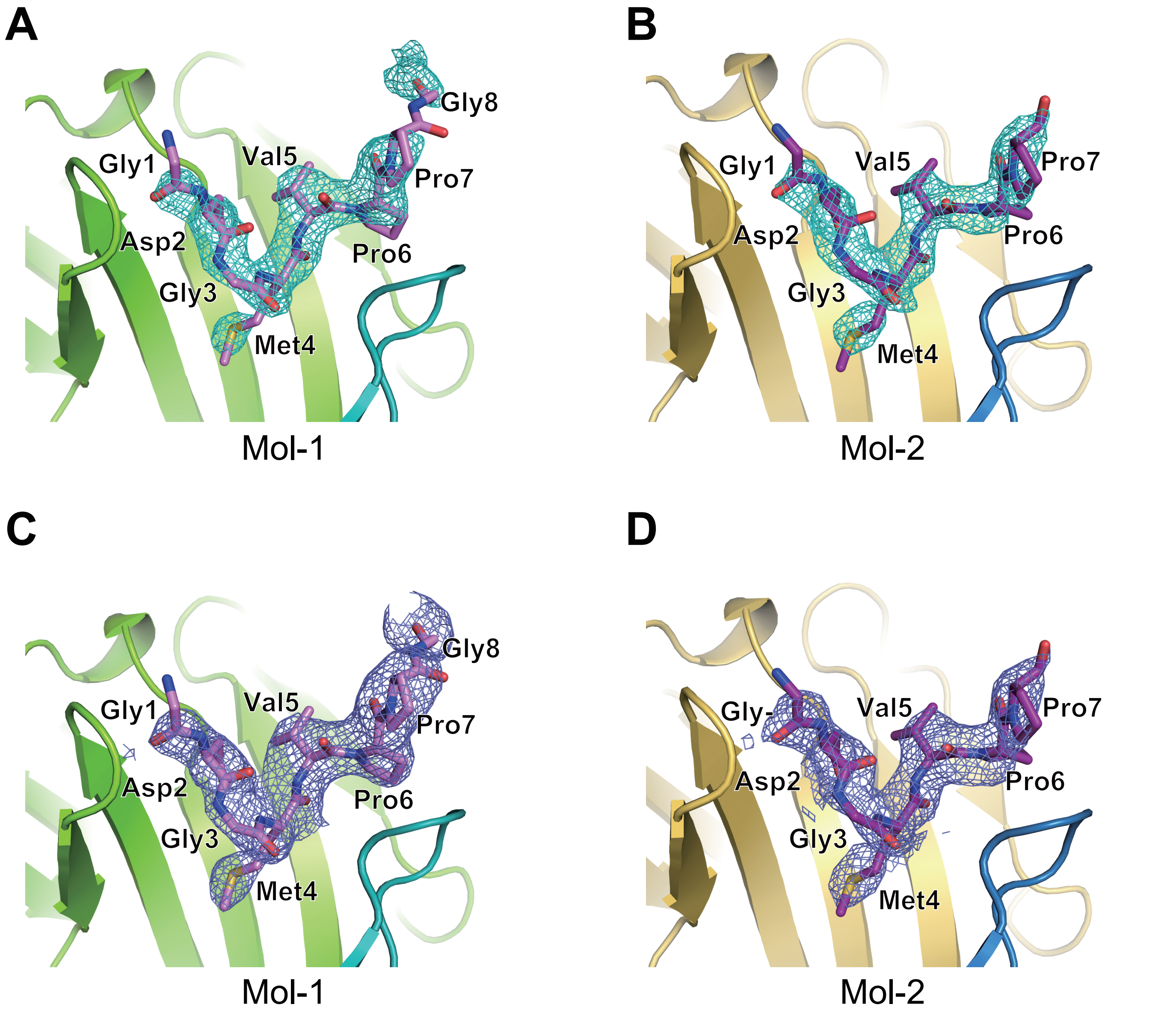

Supplementary Fig. S2 Electron density map for MAP peptides. (A and B) The *F*o-*F*c electron density map (contoured at 3.0 σ level) observed in Mol-1 (A) and Mol-2 (B) before assignment of the peptide models is colored cyan, and the 2*F*o-*F*c electron density map (contoured at 1.2 σ level) observed in Mol-1 (C) and Mol-2 (D) after the assignment is colored blue. The MAP peptides are shown as light-magenta (Mol-1) and deep-magenta (Mol-2) stick models.

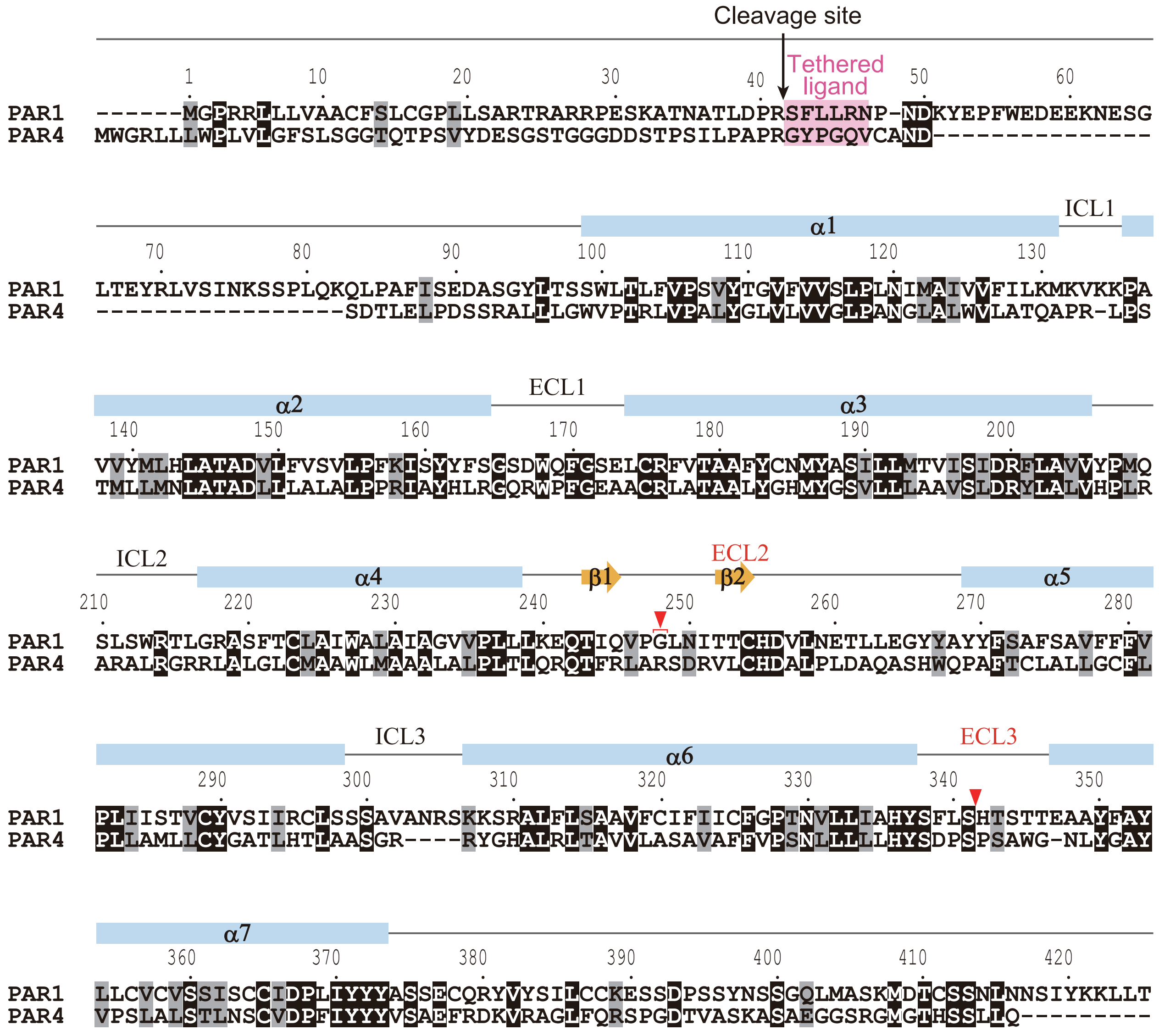

Supplementary Fig. S3 Sequence alignment of human PAR1 and human PAR4. Secondary structural elements in the PAR1 structure are shown above the sequence. Shown residue numbers are based on PAR1. The tethered ligand sequences are highlighted in pink. The MAP tag inserted points in the present study are indicated by red arrowheads.
